# Co-stimulatory agonists: An insight into the immunotherapy of cancer

**DOI:** 10.17179/excli2021-3522

**Published:** 2021-06-09

**Authors:** Ramin Pourakbari, Farnaz Hajizadeh, Forough Parhizkar, Ali Aghebati-Maleki, Sanaz Mansouri, Leili Aghebati-Maleki

**Affiliations:** 1 Stem Cell Research Center, Tabriz University of Medical Sciences, Tabriz, Iran; 2 Student's Research Committee, Tabriz University of Medical Sciences, Tabriz, Iran; 3 Department of Immunology, Tabriz University of Medical Sciences, Tabriz, Iran; 4 Drug Applied Research Center, Tabriz University of Medical Sciences, Tabriz, Iran; 5 Immunology Research Center, Tabriz University of Medical Sciences, Tabriz, Iran

**Keywords:** co-stimulatory agonists, immune checkpoint pathways, immunotherapy

## Abstract

Immune checkpoint pathways consist of stimulatory pathways, which can function like a strong impulse to promote T helper cells or killer CD8^+^ cells activation and proliferation. On the other hand, inhibitory pathways keep self-tolerance of the immune response. Increasing immunological activity by stimulating and blocking these signaling pathways are recognized as immune checkpoint therapies. Providing the best responses of CD8^+^ T cell needs the activation of T cell receptor along with the co-stimulation that is generated via stimulatory checkpoint pathways ligation including Inducible Co-Stimulator (ICOS), CD40, 4-1BB, GITR, and OX40. In cancer, programmed cell death receptor-1 (PD-1), Programmed cell death ligand-1(PD-L1) and Cytotoxic T Lymphocyte-Associated molecule-4 (CTLA-4) are the most known inhibitory checkpoint pathways, which can hinder the immune responses which have specifically anti-tumor characteristics and attenuate T cell activation and also cytokine production. The use of antagonistic monoclonal antibodies (mAbs) that block CTLA-4 or PD-1 activation is used in a variety of malignancies. It has been reported that they can lead to an increase in T cells and thereby strengthen anti-tumor immunity. Agonists of stimulatory checkpoint pathways can induce strong immunologic responses in metastatic patients; however, for achieving long-lasting benefits for the wide range of patients, efficient combinatorial therapies are required. In the present review, we focus on the preclinical and basic research on the molecular and cellular mechanisms by which immune checkpoint inhibitor blockade or other approaches with co-stimulatory agonists work together to improve T-cell antitumor immunity.

## Introduction

During recent 50 years physicians have fought with cancer mainly through surgery, radiotherapy and chemotherapy. Nevertheless, cancer still remained as a mortal disease. In more recent times, immunotherapy has opened new horizons in treating cancer (Mellman et al., 2011[64]). The immune system plays a systemic role in body's fighting against development of the tumors. Nevertheless, tumor cells can get away from body immune superintendence when changing their biological properties. The functions of immune factors in the incidence, progression, and healing of tumor has provided great interest in immunology of tumor and immunotherapy of cancer (Lisiecka and Kostro, 2016[56]; Mohme et al., 2017[66]). Several therapeutic approaches have designed to attack tumors directly. Among them immunotherapy of cancer triggers body immune function to attack cancer (Koshy and Mooney, 2016[48]; Palucka and Coussens, 2016[71]; Pourakbari et al., 2020[74]). Cancer immunotherapy after many years of progression has significantly altered treating ways of cancer. There are a number of great achievements in cancer immunotherapy including vaccines for cancers, T cells modified by Chimeric Antigen Receptor (CAR), and blocking immune checkpoint which are greatly developed by clinical trials (Moynihan et al., 2016[68]; Faltas and Tagawa, 2017[18]; Ribas and Wolchok, 2018[78]).

**Figure 1:**
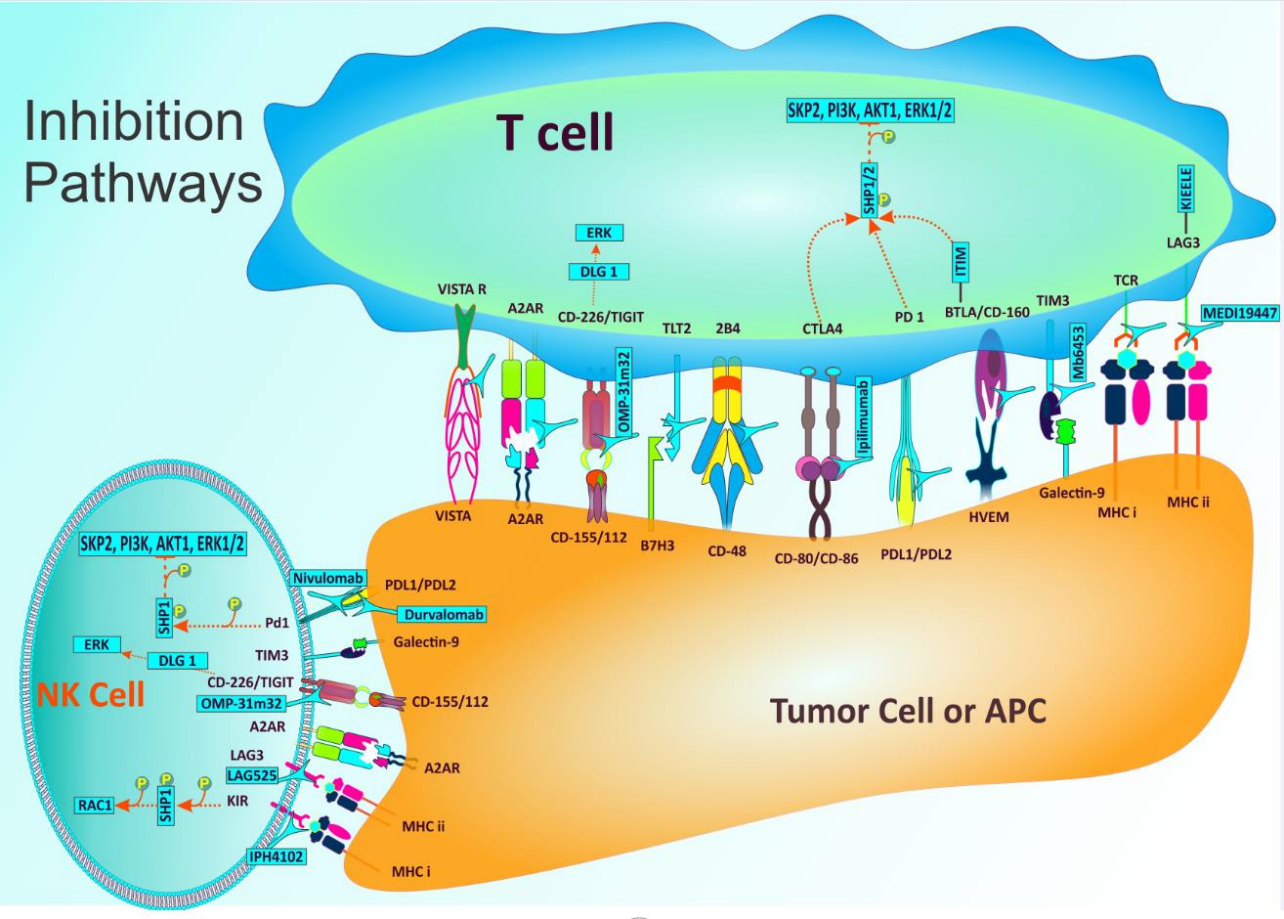
Important mechanisms identified to date that are involved in immune checkpoint inhibitory pathways

The competition between inhibitory and stimulatory signals is the origin of host immune response against tumor. As the main immune regulators, immune checkpoints maintain immune homeostasis and prevent autoimmunity. This process includes both stimulatory and inhibitory pathways which have fundamental function in preserving self-tolerance and regulating the type, magnitude, and duration of immune response (Yao et al., 2013[102]). In normal conditions, immune checkpoints permit the immune system to fight against malignancies and infectious disorders and at the same time protect tissues from any damage that can be originated from this action (Figure 1(Fig. 1)). Nevertheless, malignant cells express some of these immune-checkpoint proteins that contribute in the dysregulation of antitumor immunity and supports cancer cells to growth and expansion. Immune checkpoint therapy in the field of cancer covers those approaches that target these regulatory pathways aiming at enhancing the activity of immune system to confront tumor cells. The checkpoints that are most widely investigated are inhibitory pathways including CTLA-4, PD-1, and PD-L1 (He et al., 2015[31]; Goodman et al., 2017[25]). In 2011, Ipilimumab [anti-CTLA-4 monoclonal antibody (mAb)] was the first Immune Checkpoint Inhibitor that gained approval by the American Food and Drug Administration (FDA). Multiple biological factors which have the potential of targeting these types of molecules are extensively utilized to fight against different malignancies (Kyi and Postow, 2016[52]; Sadreddini et al., 2019[82]).

**Figure 2:**
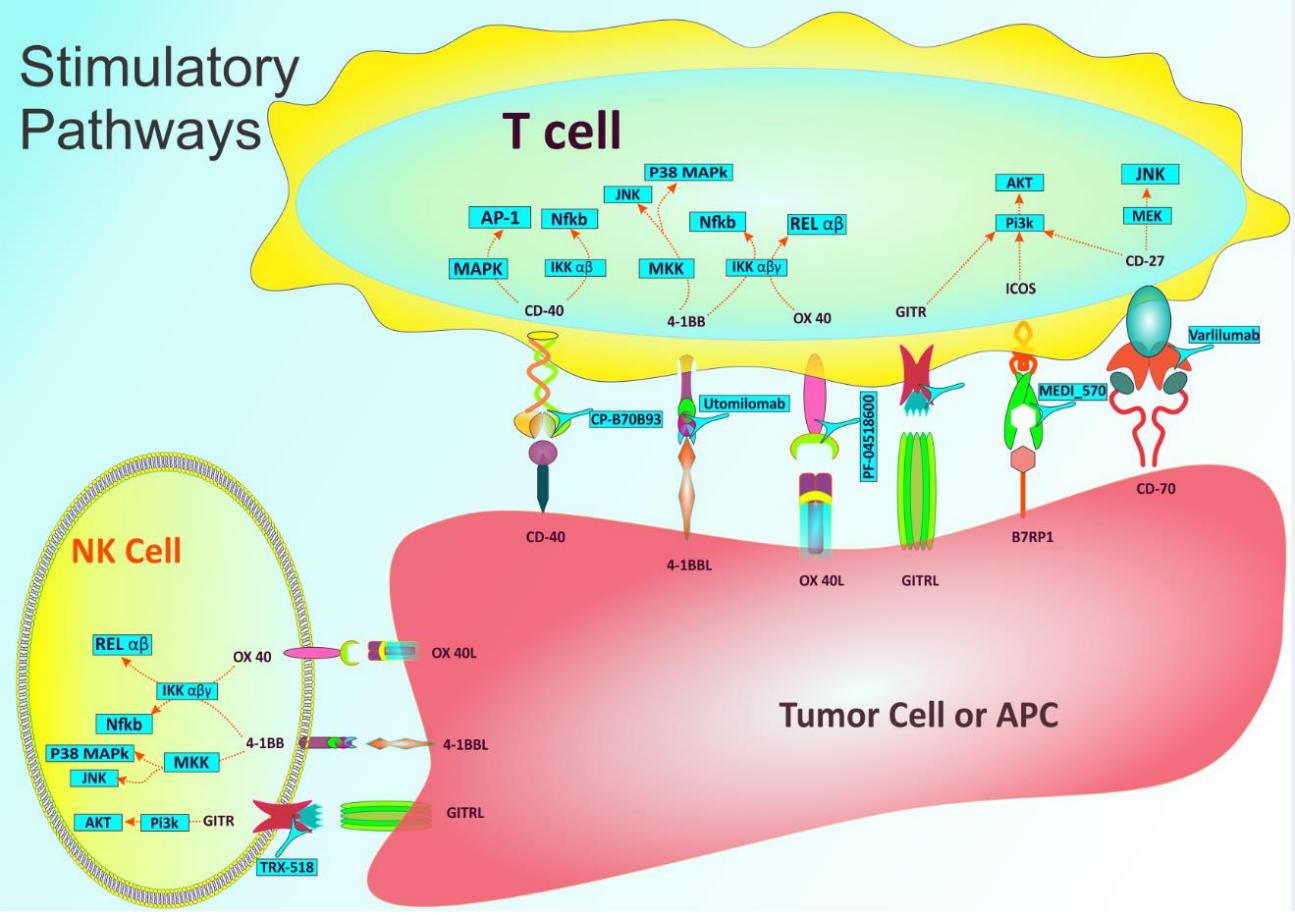
Important mechanisms identified to date that are involved in co-stimulatory pathways

Co-stimulatory molecules enhance the level of immunological responses against malignant cells, in contrary to inhibitory pathways that weaken the immune system (Figure 2(Fig. 2)). To promote tumorigenesis, malignant cells inhibit these kinds of pathways. There are a number of co-stimulatory molecules such as CD27 and CD28 which are basically expressed on resting antigen-naive T cells. Others such as ICOS, CD137, OX40, and GITR only exist on the membrane when the lymphocytes are antigen-primed or increase their expression from very low baseline levels, this point is a distinctive specification of immunotherapy whereas some co-stimulatory receptors can potentially act throughout the priming meanwhile other co-stimulatory functions solely happen on those T cells being recently primed. It must be noted that these kinds of interactions almost happen in the base of cell-to-cell interactions called as immune synapses being organized through the actions of adhesion molecules (integrins and their ligand) (Fooksman et al., 2009[21]; Melero et al., 2018[63]).

In this article, we have discussed the steps taken to develop more efficient immunotherapy strategies for cancer patients. In specific terms, we have reviewed the clinical and immunological findings gained by utilizing co-stimulatory agonists as a guiding way to implement this strategy into more efficient combinations based on two main actions: Lowering down the burden of tumor (direct antitumor impacts) and increasing the immunogenicity of tumor (indirect anti-tumor impacts mediated by immune system).

## Stimulatory Checkpoints and their Agonistic Antibodies

### OX40

OX40 (CD134 or TNFRSF4 are its other names) transmembrane protein type I that belongs to TNFR family and its expression happens on both CD4+/CD8+ T cells (at lower levels in the latter) 24-72 h after T cell receptor (TCR) engagement (Paterson et al., 1987[72]). Treating with agonist anti-OX40 mAbs accompanied by the stimulation of TCR in wild animal models caused CD4/CD8 T cells expansion and differentiation and also increased their survival. OX40 expression happened after TCR/CD3 cross-linking and in the presence of inflammatory cytokines like interleukin (IL)-1, IL-2, and tumor necrosis factor-alpha (TNF-α) (Mallett et al., 1990[58]; Croft, 2009[16]). In mice, T regulatory (Treg) cells constitutively express OX40, but in human Treg cells, the expression of OX40 is up-regulated by its activation. Signaling of OX40 inhibits the production of IL-10 and Tregs suppressive function. Another indication was that administration of anti-OX40-mAbs before engraftment of the tumor rendered Tregs functionally inactive by inhibiting IL-10 production and eliminating Treg-mediated suppression of the CD8 T-cell response. Other cells such as Natural Killer (NK) cells, neutrophils or NKT cells can also express OX40 (all belonging to the innate immune system) (Baumann et al., 2004[5]). Pro-inflammatory and pro-survival effects have been observed by the stimulation of these cells via OX40. OX40L and CD252 (as OX40 ligand) to the great extent are expressed on activated APCs, however other hematopoietic like Natural Killer cells, mast cells, activated T cells and non-hematopoietic cells including vascular endothelial cells and smooth muscle cells can similarly express OX40L (Baum et al., 1994[4]; Godfrey et al., 1994[24]). OX40 immunotherapy, utilizing agonist mAbs, is efficient to eliminate immunogenic tumors such as CT26 colon carcinoma, MC303 sarcoma, SM1 BRCA, and B16 melanoma of preclinical tumor models (Table 1(Tab. 1); References in Table 1: Bae et al., 2019[3]; Fear et al., 2018[20]; Fromm et al., 2018[23]; Guo et al., 2014[28]; Hirschhorn-Cymerman et al., 2009[33]; Houot and Levy, 2009[35]; Jahan et al., 2018[37], 2019[36]; Kinkead et al., 2018[45]; Kvarnhammar et al., 2019[51]; Linch et al., 2016[55]; Malamas et al., 2017[57]; Messenheimer et al., 2017[65]; Niknam et al., 2018[70]; Redmond et al., 2014[77]; Shrimali et al., 2017[87]; Yokouchi et al., 2008[103]). However, OX40 did not show satisfactory antitumor immunity in those tumors with poor immunogenicity. Thus, various combinatorial strategies were investigated to enhance antitumor efficacy of OX40 agonist (Kjærgaard et al., 2000[46]; Weinberg et al., 2000[100]; Redmond et al., 2009[76]).

#### PD-1 blockade with OX40 agonism

Parallel to this, Zhiqiang Guo et al. investigated effects and antitumor mechanisms of combinatorial PD-1 blockade and the trigger of OX40 in ID8 ovarian cancer mice model. They showed that this combination notably enhanced the ratio of CD8 T cells where the tumor was placed (peritoneal cavity). They concluded that it was associated to both Myeloid-Derived Suppressor Cells (MDSCs) and Tregs. Moreover, when the anti-OX40 was combined with anti-PD-1 mAbs it significantly promoted the peritoneal CD4 and CD8 effector memory cells and simultaneously reduced naïve T cells frequency. It was also reported that T cells residing in tumor- and under anti-PD1/anti-OX40 treatment notably caused higher levels of IFN-Ƴ after stimulation with PMA. Moreover, splenocytes taken from this model revealed augmented re-activity toward mesothelin (an ID8-specific antigen). The researchers also found that there was synergy between PD-1 blockade and agonistic anti-OX40 to augment implantable ovarian cancer regression in mice (Guo et al., 2014[28]). Another study revealed that, in glioma-bearing mice, a triple combination therapy, consisting anti-PD-1 immunotherapy and GVAX (a Granulocyte-macrophage colony-stimulating factor (GM-CSF)-secreting allogeneic vaccine), or OX40 agonist immuno-therapy provided more survival rate when compared to each of these agents separately. Higher systemic elaboration of Th1 cytokines, more enhanced ratio of CD4+/CD8+ inside the tumor, and notably lower percent of Tregs inside CD4+intratumoral population (Jahan et al., 2019[36]) were achieved by this triple combination immunotherapy. Fromm et al. produced a human fusion protein with two sides by implementing Extra Cellular Domains (ECD) of PD1 and OX40L, bounded by a central Fc domain, and called it PD1-Fc-OX40L. It was noticed that PD1-Fc-OX40L was concentrated in the immune synapse that in turn increased T cells proliferation, IL-2, Interferon γ (IFNγ), and TNFα production. Altogether, PD1-Fc-OX40L could be purified and is depicted to bind PD-L1/L2 and OX40 at the same time and with high affinity. *In vitro* experiments have shown that PD1-Fc-OX40L functionally activated the T cells in both human and mouse models, and it significantly performed better than the blockade of PD-1/L1, OX40 agonist, or combinative form of these antibodies. Whenever the two separate antibodies, that target PD-1(L1) and OX40, being used by i.p. or *i.v*. infusion, each immediately distributed after the infusion. (Fromm et al., 2018[23]). A point to be noted is that simultaneous addition of anti-PD-1 to anti-OX40 negated OX40 Ab antitumor properties. Adding anti-PD-1 at the initial steps of therapy showed detrimental impact on the positive outcome of Ab (anti-OX40 agonist). The authors suggested that infiltration of CD8+ T-cells specific for antigen were diminished in the tumor causing poor anti-tumor response and survival. Despite that an increase in IFNγ producing E7-specifc CD8+ T cells was observed in spleen samples taken from the mouse being cured with the combinative form of anti-OX40/vaccine and PD-1 blockade, both in periphery and the tumor, these cells became apoptotic (Shrimali et al., 2017[87]). Thus, in a number of tumors, the sequence and time of treatment with antibodies that targets inhibitory and co-stimulatory receptors is fundamental to achieve success in the approach of combination therapy. Messenheimer et al. suggested rationale firm evidence that postponed PD-1 or PD-L1 blockade after co-stimulation enhanced the T cells specific for tumors, up to the level in which checkpoints can inhibit anti-tumor responses. Anti-OX40 sequential combination preceding anti-PD-1 (not reverse) caused notable promotions in therapeutic efficiency of approaches (Messenheimer et al., 2017[65]). 

#### CTLA-4 blockade with OX40 agonism

As other negative regulatory surface molecule that exists on T cells, CTLA-4 hinders co-stimulatory pathway of CD28 in competitive manner through binding to B7-1 and B7-2. Ipilimumab is a CTLA-4 blocking antibody and has been reported to have great and durable impacts. Nevertheless, the monotherapy using this agent has restricted therapeutic benefits specially when fighting against poorly immunogenic murine tumors. New reports have suggested that combination of anti-OX40/anti-CTLA-4 mAb greatly promoted survival rate in defectively immunogenic TRAMP-C1 murine prostate tumor cells and strong effector responses of CD4 and CD8 cells required to induce tumor regression (Redmond et al., 2014[77]). Fear et al. indicated that when anti-CTLA-4 and anti-OX40 were combined, it possessed synergistic properties that increased tumor regressions. Those Tregs that reside in tumors can co-express enhanced levels of GITR, OX40, and CTLA-4 relevant to the T effector subsets. The mentioned receptors, in fact, are co-expressed on great number of cells. When CTLA-4, OX40 or GITR are individually targeted, it generates efficient responses against mesothelioma. In Immune Checkpoint Blockade (ICPB)-treated mice, anti-CTLA-4^+ ^anti-OX40-based treatments induce powerful and durable responses in orthotopic and subcutaneous mesothelioma tumor model. In subcutaneous examples, reduced number of Tregs that resided in tumors and enhanced proliferation and activation of CD8 TILs were followed by complete tumor regression. Altogether, these findings suggest that, in animal models, ICPB combination can synergistically induce the powerful and lasting immunity against mesothelioma (Fear et al., 2018[20]). Kvarnhammar et al. worked on a human CTLA-4 x OX40 bi-specific IgG1 antibody (ATOR-1015) that possessed twofold modes of function including the Tregs depletion and effector T cells activation in tumor. The researchers concluded that treatments with ATOR-1015 induced anti-tumor responses and promoted survival rate in a number of syngeneic tumor models. This response was tumor-specific and made a prolonged immunological memory in treated mice. In addition, they revealed that, in mouse tumor models, ATOR1015 was localized to the tumor where it reduced Tregs frequency and increased CD8+ T cells number and their activation. Finally, ATOR-1015 increased the level of the anti-tumor response to anti-PD-1 treatment (Kvarnhammar et al., 2019[51]).

#### Vaccination

When combined with cancer vaccines, chemotherapy, or radiation, the OX40 agonist improved control of established tumors and increased the proliferation and survival of tumor-specific T cells. Different vaccination strategies have been designed in combination with co-stimulatory molecules to make anti-tumor immune responses more efficient. Parallel to this finding, Houot and Levy explained that the combination of anti-OX40 and anti-CTLA4 (that increased the activation and blocked the negative regulatory circuits which intrinsically happen in T-cells) increased the effectiveness of CpG vaccination. When intra-tumoral CpG was combined with immunomodulatory T-cell antibodies it resulted in antitumor CD8 and CD4 T-cell immunity that, without the need for chemotherapy, treated massive and systemic lymphoma tumors, and resulted in permanent immunity against tumor relapse (Houot and Levy, 2009[35]). Linch et al. proposed that in animals that were immunized by anti-DEC-205/ HER2 mAb and adjuvant [poly(I:C)] and also received anti-OX40/anti-CTLA-4 combination therapy, T helper (Th) 2-cytokine by the production of CD4 cells became limited (which solely was related to combination therapy), CD8 and CD4 cells produced more IFNγ. They also found that following the treatment, CD8 and CD4 T cells produced more MIP-1α (Macrophage Inflammatory Protein-1α)/CCL3 [chemokine (C-C motif) ligand 3], MIP-1β/CCL4, RANTES (regulated on activation, normal T-cell expressed and secreted)/CCL5, and GM-CSF. Moreover, they revealed that this therapeutic approach was linked to the comprehensive tumor subversion and infiltration of T-cells into the tumor (Linch et al., 2016[55]). It has been reported that Neoantigen-targeted vaccines cause T cell responses in immunogenic tumors like melanomas, after vaccination the response to checkpoint inhibition is improved. Kinkead et al. established that the combination of anti-PD-1, pancreatic adenocarcinoma (Panc02) peptide vaccine-ADU-V16-AddaVax combination (PancVAX), and agonist OX40 showed strong antitumor immune responding and lasting tumor clearance in mice that bore Panc02. This happened through the inducement of tumor-infiltrating lymphocytes (TILs) specific vaccination, declining the threshold for the activation of T cell, and lowering exhaustion of TILs. Moreover, adding OX40 to vaccine resulted in the reduction of co-expression of T cell exhaustion markers, Lymphocyte-Activation Gene 3 (LAG-3) and PD-1 (Kinkead et al., 2018[45]). Another investigation revealed that combination immunotherapy approach utilizing GVAX and systemic agonist anti-OX40 monoclonal antibody reduced PD-1 and T-cell immunoglobulin, and mucin-domain containing-3 (TIM-3) co-expression as along with LAG-3 and PD-1. When GVAX was combined with systemic agonist anti-OX40 monoclonal antibody it enhanced Th1 CD4+ T lymphocytes percentage, declined Th2 fraction cells and reversed intracranial exhaustion T-lymphocyte. Overall, anti-OX40 immunotherapy plays an active role against intracranial glioma, and it was shown to have a kind of synergistic effect with GVAX. In mechanistic terms, vaccination and anti-OX40 immunotherapy are complementary to each other. This is more true about glioma microenvironment (Jahan et al., 2018[37]). It has been confirmed that B-cell maturation antigen (BCMA) is a key antigen related to myeloma. Bae et al. discovered and validated new native and engineered immunogenic BCMA peptides that were HLA-A2-specific. They had the capability of producing antigen-specific CD8+ CTL with anti-tumor functions against Multiple Myeloma (MM) cells and highly expressed co-stimulatory (CD40L, OX40, GITR) and activation (CD38, CD69) molecules. Also, they found that when heteroclitic BCMA72-80 specific CTL was treated with anti-LAG-3 (checkpoint inhibitor) or anti-OX40 (immune agonist) it showed a promoted immune function particularly via central memory CTL. These findings provided the basis for clinical implementation of heteroclitic BCMA72-80 peptide, either in single form or when combined with anti-OX40 and/or anti-LAG3 therapy, in adoptive immunotherapeutic and/or vaccination approaches in providing prolonged anti-tumor immunity in MM patients or other cases with BCMA expression (Bae et al., 2019[3]). Malamas et al. reported that in opposite to the treatments with single agent, OX40L Fusion Protein (OX40L-FP) was combined with a cancer vaccine based on poxvirus (MVA-Twist-TRICOM) ant it notably declined colonies of metastasis in lung and extended survival rate. T-cells' total amount in the population of CD4+Foxp3- and effector and central memory subsets of CD4+ in spleen, lung, and draining lymph node was increased by combination therapy. It also increased CD4+ T-cells infiltration into lung metastatic zones, and enhanced functional CD8+ T-cells number being capable of producing TNFα and IFNγ. Furthermore, OX40LFP, when combined with vaccine, caused higher CD4+ and CD8+ Twist-specific responses (Malamas et al., 2017[57]).

#### Chemotherapy and radiotherapy

Except vaccination, and checkpoint inhibition there are several other therapeutic approaches which have been found to play a complementary role for the stimulation of OX40 anti-tumor activity, such as radiotherapy, chemotherapeutics, and others. An investigation found out that initial-stage tumors with greater occurrence degree of infiltration of OX40 immune cells were highly sensitive to chemotherapy. These tumors showed postponed tumor recurrence in a period of initial 6 months after the completion of chemotherapy which was done by platinum. This study showed, in a more important sense, that patients whose tumor cells had higher expression levels of OX40 receptors had better recurrence-free survival (RFS) and showed increased sensitivity to chemotherapy when their cancer recurred (Ramser et al., 2018[75]). Another research by Hirschhorn-Cymerman et al. studied a combinative form of anti-OX40 mAbs with the chemotherapeutic cyclophosphamide (famous for the activation of tumor-reactive T cells and selective depletion of Tregs). In B16 melanoma model with poor immunogenicity, the tumor regression was initiated by this combination and it induced a T cells' powerful anti-tumor response (Hirschhorn-Cymerman et al., 2009[33]). Other investigation scrutinized the application of anti-OX40 surgical resection or tumor radiation. Anti-OX40 mAb, when combined by radiotherapy, extended survival time and was more efficient than single treatment against developed tumors (Yokouchi et al., 2008[103]). Niknam et al. (2018[70]) found that radiotherapy (XRT) together with OX40 stimulation in tumors from murine model of anti-PD1-resistant lung cancer efficiently hampered systemic and local anti-tumor growth, lung metastases, and increased survival. The expansion of CD4+ and CD8+ T cells was increased by this treatment. In tumors and spleens, the expression of OX40 on T cells was induced by XRT. XRT also promoted the percentages of splenic CD103+ dendritic cells (DCs). In fact, this study provided a rational therapeutic strategy and sequence fight against anti-PD1 resistant, poorly immunogenic tumors (Ramser et al., 2018[75]).

## 4-1BB

Originally, CD137 (4-1BB or TNFSR9) can be found on activated T lymphocytes and supports IL-2 production by T cells. CD137L is the only identified transmembrane ligand and belongs to the TNF family (Pollok et al., 1993[73]). It has been illustrated that different Antigen-Presenting Cells (APCs) such as monocytes, Dendritic Cells, and activated B cells can express 4‐1BBL. In opposite, 4-1BB can be initially expressed on activated T cells and NK cells (Melero et al., 1998[61], 2008[62]). Preliminary investigations found that 4-1BB ligation by cell-surface 4-1BBL or 4-1BB-specific mAb made powerful co-stimulatory signals to T cells that included both CD4+ and CD8+ T cells, and increased the survival rate of T cells *in vitro* and *in vivo* that is related to the Bcl-XL and Bfl-1increased intracellular levels (Vinay and Kwon 2011[97]; Vinay et al., 2004[96]). The therapeutic effects were originated by agonist mAbs and mediated by potent CTL response which effectively eliminate the malignancies (Melero et al., 1998[61]). In highly-resistant tumors combination strategies with other therapies which finally cause synergistic and often curative effects are easy to find and accessible (Shi and Siemann, 2006[85]). These strategies can be a variety of combinations with cytokines, vaccines, and other immune-stimulatory mAbs. Furthermore, it has been reported that both radiotherapy and chemotherapy are synergistic with anti-CD137 mAb (Table 2(Tab. 2); References in Table 2: Azpilikueta et al., 2016[2]; Belcaid et al., 2014[6]; Buchan et al., 2018[9]; Chen et al., 2015[13]; Curran et al., 2011[17]; Guillerey et al., 2019[26]; Hebb et al., 2018[32]; Hosoi et al., 2018[34]; Jang et al., 2018[38]; Jensen et al., 2013[39]; Ju et al., 2008[40]; Kerage et al., 2018[42]; Kim et al., 2009[44], 2013[43]; Kobayashi et al., 2015[47]; Kosmides et al., 2017[49]; Kroon et al., 2016[50]; Läubli et al., 2018[53]; Lee et al., 2011[54]; McKee et al., 2017[60]; Morales-Kastresana et al., 2013[67]; Newcomb et al., 2010[69]; Redmond et al., 2014[77]; Rodriguez-Ruiz et al., 2016[80], 2017[79]; Shi and Siemann, 2006[85]; Shindo et al., 2015[86]; Sin et al., 2013[88]; Tongu et al., 2015[91]; Verbrugge et al., 2012[94]; Youlin et al., 2012[104]).

### PD-1 blockade with 4-1BB agonism

Accordingly, Shindo et al. studied the combinative form of mAb against 4-1BB as a co-stimulatory effector and PD-1 as a blockade of the immune checkpoint. Anti-4-1BB's anti-tumor impact probably is related to the increased activity of tumor-specific cytotoxic T lymphocyte and the production of IFN-γ through CD4+ and CD8+ T-cells. Furthermore, in all mice, this therapeutic approach caused high number of CD4+ IFN-γ+ T-cells (Th1 cells) and CD8+ IFN-γ+ cells contributing to the full rejection of tumor (Shindo et al., 2015[86]). Azpilikueta et al. studied the combinative form of anti-PD-1/PD-L1 with anti-CD137 mAb immunotherapy to fight squamous non-small cell lung cancer. Therapies utilizing single agent did not have enough efficiency, nevertheless, the combinative form of anti-PD-1 and anti CD137 resulted in complete rejections. Efficacy of combined treatment needed CD8 T cells and it caused a leukocyte infiltration in which T lymphocytes co-expressed CD137 and PD-1 was in majority (Azpilikueta et al., 2016[2]). Chen et al. suggested that when anti-4-1BB was combined with anti-PD-1, it synergistically inhibited MC38 colon carcinoma and B16F10 melanoma growth in syngeneic C57BL/6 mice. Solely in those animals who received anti-4-1BB and anti-PD-1 synchronously, the tumor inhibition occurred. But when anti-LAG-3 was combined with anti-PD-1it caused moderate tumor suppression. The activity of combinative form of anti-4-1BB and anti-PD-1 depended on CD8+T and IFNγ cells, in the spleen. The immune system was shaped by the combination treatment to a memory/effector phenotype and it augmented the total performance of tumor-specific CD8+CTLs that reflected the prolonged systemic antitumor response. When the cancer vaccine at any kind is absent, anti-PD-1 combined with anti-4-1BB is efficient to make a potent antitumor memory/effector T-cell response facing invasive tumor that makes it a suitable candidate for combination trials on patients (Chen et al., 2015[13]). Hosoi et al. claimed that the suppression of tumor growth was attained solely through anti-PD-1 or when it was combined with the agonistic antibody anti-4-1BB, or with anti-CD4 mAb monotherapy. In opposite, in challenging B16 melanoma model, the tumor progressively developed in mice which were treated with anti-4-1BB mAb or anti-CTLA-4 monotherapy. To have a satisfactory immunotherapy in its objectives, a proper combination strategy enhances T-cell variety in tumor by remaining the peripheral repertoire to be unaffected (Hosoi et al., 2018[34]). Morales-Kastresana et al. explained that triple combination approach consisting of anti-OX40 and Immunostimulatory monoclonal Antibodies (ISmAb) anti-CD137 and anti-B7-H1 (PD-L1) promoted survival rate among mice that carried hepatocellular carcinomas in a CD8-dependent fashion and were synergized with adoptive T-cell therapeutic strategy that utilized activated OVA-specific TCR transgenic OT-1 and OT-2 lymphocytes. Those mice that underwent this therapeutic approach had obviously higher infiltration of tumor by means of blastic and activated CD4+T and CD8+ lymphocytes that included perforin/granzyme B and expressed ISmAb-targeted receptors on their surface (Morales-Kastresana et al., 2013[67]). Tregs infiltrating murine or human tumors expressed great number of 4-1BB. Anti-4-1BB mAbs selectively depleted intra-tumoral Tregs *in vivo*. Effector T cell agonist was also promoted by anti-4-1BB mAbs to enhance the rejection of tumors. These distinguished processes were in competitive form and depended on the availability of FcgR and antibody isotype. Implementation of anti-4-1BB IgG2a that selectively depletes Tregs, together with either anti-PD-1 mAb or agonistic anti-4-1BB IgG1 enhanced anti-tumor responses in several solid tumors. An antibody that was made to improve both FcγR-independent agonism and FcγR-dependent Treg cell depleting capacity provided an efficient anti-tumor therapeutic approach (Buchan et al., 2018[9]). Kosmides et al. studied a kind of nanoparticle platform that overcame the immunosuppressive tumor microenvironment (TME). Two different antibodies coat these nanoparticles that concomitantly blocked the signal made by inhibitory checkpoint PD-L1 and stimulated T cells through 4-1BB co-stimulatory pathway. In several *in vivo* models of murine colon and melanoma cancer these “immunoswitch” particles led to a notable postpone in tumor growth and extension of survival, in comparison to the application of nanoparticles or soluble antibodies separately conjugated with the inhibitory and stimulating antibodies. Applying immunoswitch nanoparticles led to the enhanced specificity, functionality, and density of tumor-infiltrating CD8+ T cells *in vivo*. Alterations in T cell receptor repertoire against a single tumor antigen suggested that immunoswitch particles extended an efficient set of T cell clones (Kosmides et al., 2017[49]). 

### CTLA-4 blockade with 4-1BB agonism

CTLA-4 (a co-inhibitory receptor) declines immune responses and prevents autoimmunity, nevertheless, tumors use it when they attack host T cell response. CTLA-4 blocking or 4-1BB activating antibodies can increase some murine tumors rejection, but they are unsuccessful to treat tumors with poor immunogenicity such as B16 melanoma as single factors. Curran et al. found that the activation of 4-1BB enhanced infiltration of CD8 in tumors, production of inflammatory cytokine by peripheral CD8 cells, and more strongly proliferation of tumor infiltrating CD8^+ ^T cells. Moreover, anti-4-1BB led to the polarization of cytokine production into TH1 and also induced the TNF-α production by CD8 T-cells and IFN-γ production by CD4 cells above the sum of each single therapeutic approach. It was while; the infiltration and proliferation of CD4 effector were only promoted by CTLA-4 blockade. Activation of CD8 T-cells by α4-1BB along with the expansion of CD4 effector T-cells by anti-CTLA-4 obviously suggested the noticed synergy between these agents to reject B16 melanomas. Α 4-1BB attenuated proliferation of Tregs, dampens the Tregs fraction of TIL, counteracts anti-CTLA4's expansion of absolute numbers of Treg in the tumor, and declines CTLA-4 and PD-1 expression by Tregs. Α 4-1BB caused a significant up-regulation of killer cell lectin-like receptor G1 (KLRG1) on CD8^+^, and less extent the up-regulation of effector T-cells and CD4+ in the tumor. This up-regulation was apparently unique for 4-1BB agonist antibody, because the same phenotype, in response to anti-CTLA-4, anti-PD-1, or anti-PD-L1, was not observed. It was found that mice that received the therapeutically efficient combination of anti-CTLA-4/anti-4-1BB possessed 1.7 times more CD4^+^KLRG1^+^ cells that infiltrated tumors when compared with the mice being treated only with anti-4-1BB which suggest probable functional importance to this population. Furthermore, KLRG1 expression of CD8 and CD4 effector cells is increased in TIL over the time of the treatment. It suggests either promoted infiltration, survival, or proliferation rate of these cells. This research depicted that combination of T-cell co-inhibitory blockade with anti-CTLA-4, and active co-stimulation with anti-4-1BB promoted rejection of B16 melanoma if a suitable vaccine is provided (Curran et al., 2011[17]). When anti-CTLA-4 is combined with anti-4-1BB antibodies enhance it increased tumor immunity. However, for all tumors, the approach does not have enough efficiency, and it is proposed that differences in tumor control can show the variations in the immunogenicity of diverse tumors. The magnitude of T-cell repertoire that targets tumor has a key role in demonstrating the effectiveness of this therapeutic approach. More specifically, combination strategy was completely unsuccessful to hamper the GP-expressing tumor cells in the mice expressing the exogenous antigen as a self-antigen and carrying a severely purged T-cell repertoire directed against the major tumor antigen. It highlights the significance of T-cell population that are intact functionally as a prerequisite for achieving a satisfactory level of efficiency of immunomodulatory antibodies-based treatments. This type of therapy must be tried as an initial type of tumor-specific immunotherapy former too wide exhaustion of the tumor-specific T-cell repertoire would happen (Jensen et al., 2013[39]). Hebb et al. explained that peri-tumor draining lymph node (tDLN) or intertumoral administration of the newly discovered combinative form of anti-CD137, anti-CTLA4, and anti-OX40 acquired powerful systemic anti-tumor properties. This combination was applied intratumorally at low doses on one tumor of a mouse model with two tumors. It left significant systemic and local anti-tumor effects on lymphoma (A20) and solid tumor (MC38) models. This investigation suggested that the intratumoral implementation anti-CD137, anti-OX40, and anti-CTLA4 10 µg in combinative form was more efficient than the systemic application which supports the concept of* in situ* vaccine. The possibility of using these low doses in comparison with the typical doses of 100-400 µg for these agents, is a fundamental issue in decreasing the toxicity level. Therefore, as explored in the project, at the highest doses, the toxicity was noticed, but at medium and the lower doses, the triple combination therapy was safe with relative hematological toxicity. The triple combination caused bilateral regression of tumor in MC38 and A20 tumors; nevertheless, it was highly efficient in the A20. Several potential variables cause the differences in the efficacy such as immunogenicity and growth kinetics of the tumor cells, microenvironments' intrinsic variations, T-cell repertoire, and variations in immune responses between the two mouse strains (Hebb et al., 2018[32]). Youlin et al. evaluated the effect of 4-1BBL-expressing tumor cell vaccine (in comparison to the CTLA-4 blockade) on murine prostate cancer RM-1 rejection. When compared to the treatment by each of these agents alone, it resulted in RM-1 tumors regression and survival of the tumor cell recipients was significantly increased. The combined vaccination caused higher number of CTL against RM-1 cells and also enhanced IL-2, IFN-γ, and TNF-α, secretion in the mix-cultured supernatant (Youlin et al., 2012[104]). In total, all of these suggest that the combination of 4-1BB and CTLA-4 blockade can be a promising approach in cancer immunotherapy.

### Other inhibitory molecules

T-cell Immunoglobulin and Mucin domain 3 (TIM-3) is famous for being a negative regulator in immune system. According to the recent findings, TIM-3 possesses a fundamental function in the suppression of antitumor immunity. Guo et al. suggested that the combinative form of TIM-3 blockade and the activation of CD137 notably enhanced immunotherapy in the ovarian cancer of murine ID8 model. Either CD137 mAb or anti-TIM-3 in single form, despite being efficient in 3-days tumor, were failed in hampering tumor progression in mice having 10-days established tumor. However, the combination of CD137 mAb and anti-TIM-3 severely limited tumor growth with 60 % of tumor-free mice 90 days after the tumor was inoculated. The combination of 2 mAbs significantly increased CD8+ and CD4+ cells and decreased immunosuppressive CD4+FoxP3+ Tregs and CD11b+Gr-1+ Myeloid Suppressor Cells (MDSC) at tumor sites. This supports directing the responses of local immune system toward an immunostimulatory Th1 type. This finding is additionally supported by RT-PCR quantitative data suggesting enhancement of Th1-associated genes by the treatment with anti-TIM-3/CD137. Elevated number of CD8+ T cells originated great amounts of IFN-γ by the stimulation of tumor antigen and showed antigen-specific cytotoxic activity. The combinative form of TIM-3 blockade and CD137 activation synergistically caused powerful antitumor impacts in ID8 ovarian cancer model. This finding can help to design the future trials for the immunotherapy of ovarian cancer (Guo et al., 2013[27]). 

### Tumor vaccine 

Recent investigations have found that when modulatory immune strategies are combined with each other it can help to the elimination of tumor cells. Most approaches have emphasized the utilization of monoclonal antibodies being able to block receptors on cell surfaces to reduce immunosuppression induced by tumors or act as co-stimulatory ligands to increase T cells' activation. Manrique-Rincón et al. studied the application of the genetically modified cell lines derived from tumors in combinative form that were harboring the co-stimulatory T cell ligands 4-1BB ligand, OX40L, and the cytokine GM-CSF. These cells that are derived from tumors can either activate or reinforce T cell activation and thus, resulting in a powerful and specific antitumor response. Combining tumor-derived cells expressing co-stimulatory ligands with GM-CSF caused prolonged protective impact by preventing from cancer progression in both treated and re-challenged animals. These results indicate that it is highly beneficial to use combined forms of syngeneic tumor vaccines that express immunomodulators (Manrique-Rincón et al., 2017[59]). Autologous tumor cell vaccination strategies using synthetic glycolipid immune adjuvant, α-galactosylceramide (α-GalCer), targeting the immunoregulatory properties of NKT cells are suggested to be efficient in preventing from tumor progression by enhancing NK cells and T cells generation and activities. Kobayashi et al. reported that B cell lymphoma therapeutic anticancer vaccination using NKT cell ligand can be enhanced by the following co-stimulation through 4-1BB that results in a long-lasting response adding more to the outcomes of conventional treatments. The α-Galactosylceramide (α-GalCer)-loaded tumor cell vaccination together with anti-4-1BB antibody caused significant enhancement in survival of those mice that harbored Eµ-myc tumors. It included full elimination of lymphoma in nearly 50 % of the samples. Tumor-free survival required IFNγ-dependent expansion of CD8+ T cells and was related to 4-1BB-mediated differentiation of KLRG1+ effector CD8+ T cells. Also, 'cured' mice resisted to lymphoma relapse, 80 days after the indication of successful generation of immunological memory in these mice (Kobayashi et al., 2015[47]). Kerage et al. studied the efficiency of agonistic anti-4-1BB combination therapy based on antibody to treat two invasive forms of Acute Myeloid Leukemia. Treatment with anti-4-1BB, when solely used, caused suppression of the progression of established AML-ETO9a tumor for a short time almost in 50 % of mice. Nevertheless, many of these mice finally surrendered to the AML. The combinative form of alpha-galactosylceramide (α-GalCer)-loaded tumor cell vaccination and anti-4-1BB antibody promoted percentage of responding mice up to one hundred. It also provided prolonged tumor-free survival that showed the full AML elimination. Overall, the combination of NKT cell-targeting vaccination and anti-4-1BB produce unique outcomes in the treatment of AML and MLL among mice (Kerage et al., 2018[42]). Combinative form of anti-4-1BB and anti-PD-1, despite that it improved survival, when compared to anti-4-1BB alone, was not as efficient as NKT cell vaccination. McKee et al. suggested that the combination of 4-1BB with α-GalCer-loaded, irradiated tumor cell vaccine, 4-1BB mAb treatment resulted in the promoted expansion of populations of effector CD8 T-cells and long-term protection of surviving mice against tumor relapse. Surprisingly, no therapeutic benefit was provided with PD-1 blockade. In case of simultaneous utilization with a PD-1-blocking mAb the T-cell-promoting effects of 4-1BB mAb and its antitumor activity are diminished. This issue is related to an immediate and severe decline in subsets of effector CD8+T-cell in the presence of PD-1 blockade. These results depict that therapeutic support of T-cell activation plays an effective role in the control of B-cell lymphomas; however, the caution must be paid when combining antibody-mediated modulation of both co-inhibitory and co-stimulatory T-cell receptors (McKee et al., 2017[60]). Kim et al. revealed that pE7+IL-2 cDNA co-delivery raised the rates of antitumor activity from 7 % to 27 %, while pE7+IL-2 cDNA co-delivery with anti-4-1BB Abs increased it from 27 % to 67 % and provided prolonged memory responses. This enhanced activity was concurrent with the enhanced induction of activity of IFN-γ and Ag-specific CTL responses, but not with the production of Ag-specific IgG. Furthermore, 4-1BB and IL-2 receptors and anti-4-1BB Abs and rIL-2 combined stimulation caused higher production of IFN-γ from Ag-specific CD8^+^ T cells. Nevertheless, this effect was diminished by anti-IL-2 Abs and 4-1BB-Fc treatment. It suggests the fact that the observed impact was specific for IL-2- and anti-4-1BB Ab. These investigations, therefore, illustrate that via IL-2 and 4-1BB receptors combined stimulation enhances pE7-induced Ag-specific CD8^+^ CTL responses that increased the rates of tumor treatment and prolonged antitumor immune memory. These results probably have merits in designing DNA-based therapeutic vaccines when fighting against cancer (Kim et al., 2013[43]). Jang et al. investigated the therapeutic properties of gemcitabine in addition to E7 peptide vaccine regimens (E7 peptides+CpG-ODN+anti-4-1BB Abs) on TC-1 tumors. They revealed that the preliminary combination therapy utilizing both gemcitabine and E7 peptide vaccine regimens caused tumor regression with tumor relapse in animals with big and developed tumors, which apparently was the result of the suppression of Ag-specific CTL activity when treated with gemcitabine. However, in all tested mice, gemcitabine therapy optimization by lowering down its dose and frequency caused tumor total regression without any tumor relapse even after the therapy was discontinued, possibly due to the Ag-specific CTL responses. Therefore, this investigation showed that gemcitabine optimal dose and its utilization frequency are fundamental in achieving tumor treatments in animals with tumors who undergo E7 peptide vaccine regimen therapy, principally through the prevention of CTL suppression (Jang et al., 2018[38]). Sin et al. reported that the treatment with Trp2 peptides and CpG-oligodeoxynucleotide induced Ag-specific IFN-γ and CD8^+^ CTL responses and caused the antitumor activities against large melanoma tumors. When anti-4-1BB antibodies were combined with Trp2 peptides and CpG-oligodeoxynucleotide, they promoted the antitumor activity from 0 % up to 75 %. It was simultaneous to the higher induction of Ag-specific CD8^+^ CTLs and their infiltration into the tumoral tissues. It shows TLR9 and 4-1BB significance in combinative form and its stimulation in eliminating tumors (Sin et al., 2013[88]). Combining DCs vaccine with 4-1BB ligation is an optimal immunotherapy alternative for irremediable cancers. Nevertheless, in anti-tumor effector doses over 100 µg, 4-1BB Ab ligation is toxic to CD4^+^ T cells and limits its treatment utilization. Lee et al. reported that CD3^+^CD8^+^T cells in the 20 µg 4-1BB ligation group, were highly induced causing no toxicity to CD3^+^CD4^+^T cells. Treatment with DC vaccine leads to the secretion of tumor antigen-specific Th1 cytokine (IL-2 and IFN-γ) from the splenic lymphocytes. 4-1BB ligation declined the secretion of IL-10 related to DC vaccine and also reduced regulatory T cell population. In comparison to the anti-tumor impact of single utilization of either 20 µg 4-1BB Ab or DC vaccine, combination therapy greatly enhanced the power of tumor rejection up to the level that was observed when higher doses of 4-1BB Ab were applied alone. The combinative form did not cause high-dose 4-1BB-related toxicity with CD4^+^T cell reduction, but it notably produced antigen-specific tumor IFN-γ which secreted effector CD8^+^ cytotoxic T cells. Here, it has been depicted that how much valuable is DC vaccine when is combined with 4-1BB Ab even at low doses (20 µg) to be an improved immunotherapeutic strategy in the field of cancer (Lee et al., 2011[54]).

### Chemotherapy

Chemotherapy can be a prerequisite of immunotherapy by providing a setting for homeostatic lymphoproliferation and by diminishing several networks that suppress immune system. The combinative form of anti-CD137 mAb immunotherapy and Tregs' depletion being utilized later to chemotherapy with cyclophosphamide or melphalan effectively declined the burden of Multiple Myeloma (MM) and extended survival (Guillerey et al., 2019[26]). Kim et al. found that when anti-4-1BB was combined with Cyclophosphamide (CP) it caused synergistic anticancer conditions in the poorly immunogenic B16 melanoma model among mice. The combination treatment antitumor impacts were principally mediated by CD8^+^ T cells and to some extent by NK cells. Further analysis showed that the responses of CD8^+^ T against tumor antigens was synergistically augmented by combinative form treatment by enhancing the population of CTLs expressing CD11c molecules on their surface. Tumor burden was declined by CP and it mainly depleted the naive T-cell compartment, while naive T cells were protected by triggering 4-1BB and it caused effector/memory and memory T cells to be expanded. It was also depicted that the peripheral T cells that survived from the CP treatment were protected and boosted by triggering 4-1BB, while the memory T cells were preferentially spared. Treatment with CP elevated the expansion of 4-1BB on CD8 T and CD4 cells and CP, either alone or in combinative form with anti-4-1BB, effectively caused the suppression of peripheral Tregs. These findings implicate that anti-CP and 4-1BB can act together in the treatment of cancer with CP to provide setting inside which anti-4-1BB enhances tumor-specific CTLs differentiation and expansion in active manner (Kim et al., 2009[44]). Tongu et al. evaluated antitumor impacts of a mixture of local injection with anti-CD137 mAb and intermittent low-dose chemotherapy applying CP and Gemcitabine (GEM) on CT26 colon carcinoma that was established subcutaneously. It was found that treatment with local anti-CD137 mAb was therapeutically inefficient in terms of late-stage tumors. The reason could be a severe elevation MDSC population at the tumor sites on day 17. Nevertheless, when GEM and CP was injected at low-dose (50 mg⁄kg) it decreased this elevation. Moreover, despite that the periodic injections of GEM and low-dose CP on days 10 and 18 caused significant suppression of tumor growth, further local injections of anti-CD137 mAb on days 19, 21, and 23 increased the therapeutic efficacy even further. Thus, this investigation showed that periodic chemotherapy accompanied by GEM and low-dose CP can synergistically act with local anti-CD137 mAb therapy. Intermittent immunochemotherapy as pre-treatment strategy can preserve a proper microenvironment for following local anti-CD137 mAb therapy. Due to the fact that after immunomodulating mAb therapy, tumor-reactive T cells are considered to be the main tumor regression effectors, intermittent immunochemotherapy can be a helpful strategy to support - anti-cancer treatments which are based on mAb (Tongu et al., 2015[91]). As reported, Renal Cell Carcinoma (RCC), a significantly fatal and hard-to treat cancer, is very non-responsive to radiotherapy and chemotherapy. As suggested, applying agonistic anti-4-1BB monoclonal antibody (mAb) regress several animal tumors however, the effects on RCC is still unclear. Ju et al. reported trivial effects on established RCC and Renca tumors, when utilizing monotherapy with anti-4-1BB mAb or the cytotoxic drug or 5-fluorouracil (5-FU), however, as they demonstrated, with the combinative form of anti-4-1BB mAb and 5-FU eliminated the tumors in more than 70 % of mice. Mice treated with combination therapy had more tumor infiltrating lymphocytes and apoptotic tumor cells in their regressing tissues of their tumors when compared to that of mice that were treated by anti-4-1BB mAb or 5-FU monotherapy. Lymphocytes population in Tumor-Draining Lymph Nodes (TDLNs) and spleens of those mice cured with combination therapy was highly augmented when compared to that of control or 5-FU monotherapy mice. Mice that were recovered by combination therapy immediately rejected tumor re-challenge that showed the establishment of prolonged tumor-specific memory (Ju et al., 2008[40]). Läubli et al. studied the immunomodulatory impacts of the multi-receptor tyrosine kinase inhibitor axitinib and its effectiveness when combined with immunotherapies. It improved anti-cancer immunity when combined with checkpoint inhibitors anti-PD-1 and anti-TIM-3 and/or CD137 agonistic antibodies via the modulation of anti-tumor immunity. More importantly, the combination therapy caused prolonged anti-cancer immunity and protected memory formation because when the tumor-free mice were re-challenged with the same tumor cell line they immediately rejected (Läubli et al., 2018[53]). 

### Radiotherapy

Evidence gained in preclinical and clinical phases have suggested that radiotherapy pro-immune impacts are synergistically elevated with immunostimulatory mAbs on both irradiated tumors and on remote and non-irradiated tumors. As reported by investigations, radiation enhanced the antitumor effect of anti-CD137 therapy. Radiotherapy is combined with anti-CD137 therapy it completely eradicated the tumor and prolonged survival rate in six mice (67 %) out of nine with established brain tumors. In brain tumors, the antitumor immunity correlated with the elevated number of Tumor-Infiltrating Lymphocytes (TILs) and it enhanced tumor-specific production of IFNγ (Newcomb et al., 2010[69]). In a study by Kroon et al. anti-PD-1 and anti-CD137 antibodies were shown to be most efficient in enhancing the delay of tumor growth in a mouse melanoma model after Stereotactic Body Radiation Therapy (SBRT). It shows that the capability of IL-2, or the combination of anti-PD-1 and anti-CTLA-4 to establish synergy with SBRT (Kroon et al., 2016[50]). When radiotherapy is combined with immunostimulatory anti-PD1 and anti-CD137 mAbs it left satisfactory outcomes on distant and non-irradiated tumors in transplanted B16OVA (melanoma), MC38 (colorectal cancer), and 4T1 (breast cancer) models. Radiotherapy caused alterations in immune infiltration in irradiated and non-irradiated lesions characterized by decline in the overall content of Tregs, effector T cells, and myeloid-derived suppressor cells, whereas in both irradiated and contralateral tumors, the effector T cells expressed more intracellular IFNγ. Significantly, CD8^+^ TILs exhibited more clear expression of CD137 and PD1, 48 hours after the irradiation, which depicted more target molecules for the corresponding mAbs (Rodriguez-Ruiz et al., 2016[80]). In another study by Rodriguez-Ruiz et al. they investigated radiotherapy abscopal effects provoked by brachytherapy methods. They reported that abscopal effects on other non-irradiated subcutaneous tumor lesions in transplanted tumors that were derived from MC38 happened only in case of brachytherapy administration in combination with immunostimulatory anti-PD1 and/or anti-CD137 mAbs (Rodriguez-Ruiz et al., 2017[79]). Verbrugge et al. found that simultaneous targeting of the co-stimulatory molecule CD137 with CD40 increased the antitumor effect of radiotherapy and increased the likelihood of rejection of subcutaneous BALB/c-derived 4T1.2 tumors. However, this novel combinative form was non-curative in mice that carried developed C57BL/6-derived AT-3 tumors. Nevertheless, when single- or low-dose fractionated radiotherapy was integrated with anti-PD-1 mAbs and anti-CD137 it was effective on all mice who had developed orthotopic AT-3 mammary tumors. The expression of CD137 on tumor-associated CD8^+^ T cells was mainly restricted to a subset that greatly expressed PD-1. These CD137^+^PD-1high CD8+ T cells which remained in AT-3 tumors that get irradiated, expressed granzyme B, Tim-3, and Ki67 and made IFN-γ *ex vivo* in reaction to Phorbol 12-Myristate 13-Acetate (PMA) and stimulation of ionomycin (Verbrugge et al., 2012[94]). Belcaid et al. concluded that when CTLA-4 blockade and 4-1BB activation are combined together, in focal radiation therapy, promoted the survival of orthotopic mouse model of glioma through a mechanism that depended on CD4^+^ T cell and originated memory that was antigen-specific. This therapy on mice promoted survival rate when compared to focal radiation therapy and immunotherapy by 4-1BB activation and CTLA-4 blockade. It caused at least 50 % prolonged tumor-free survival in treated animals models. It also made more elevated density of CD4^+^ and CD8^+^ tumor infiltrating lymphocytes. Antitumor effect of triple therapy was diminished by the depletion of CD4^+^ T cells, while CD8^+^ T cells depletion unaffected the treatment response (Belcaid et al., 2014[6]).

### CD27

CD27 is a molecule member of TNFR family. An exclusive characteristic of CD27 among other TNFR members is that it is constitutively expressed at higher levels on most T cells (mainly on naive T cells) (Van Lier et al., 1987[93]). Regarding CD27 constitutive expression pattern, the expression of CD70, as its only known ligand, is tightly regulated. Under physiological conditions, CD70, in fact, have only short-term expression on activated T cells, APC, and NK cells (Borst et al., 2005[8]). Utilizing CD27 to be a cancer immunotherapy target is complicated by the co-stimulatory and inhibitory mechanisms associated to the CD27-CD70 pathway in variety of immune settings. The ligation of CD27 through CD70 employs TNF receptor-associated factor (TRAF) 2 and TRAF5 to the intracellular domain of CD27 which activates c-Jun and NF-κβ pathways and enhances cell survival, increases the expansion of B and T cells, and enhances effector functions (Yang et al., 2007[101]). CD70 constitutive expression has been reported in the field of cancer. It is noticed that, in murine lymphoma models, the constitutive expression of CD70 either on tumors or APCs enhances antitumor immunity which in turn, promotes NK-mediated rejection (Kelly et al., 2002[41]). Parallel to this finding, it has been depicted that utilizing an agonistic anti-CD27 antibody protected against *IV* injection of two distinct lymphoma cell lines (French et al., 2007[22]). Overall, it is obvious that CD27 impacts may lie on the tissue context in which the CD70 is expressed, and also on time length of CD27-CD70 ligation. When agents that interact with CD27 are clinically developed, they will need an exact triggering of specific molecules in selected contexts to avoid the exaggeration of immunosuppression induced by tumors. CD27 is an appropriated co-stimulatory receptor, with good characteristics, for CD8^+^ T cells and when examined as a monotherapy, agonist anti-CD27 mAb was superior to mAbs targeting other co-stimulatory receptors such as OX40, 4-1BB and GITR in driving expansion of gp100-specific CD8^+^ T cells *in vivo*. Buchan et al. found that when agonist anti-CD27 was combined with anti-PD-1/L1 mAb is indeed synergistic; it improved pmel1 CD8^+^ T cells priming indicated by promoted proliferation of T-cell, exemplified by augmented TNF-α, IFN-γ, granzyme B and T-bet, and increased differentiation into effector T cells. As a result, compared to monotherapy, in some pre-clinical tumor models, the combined treatment provided stronger anti-tumor immunity. Moreover, varlilumab, which is anti-human CD27 mAb is in clinical examination phase I or II at the present moment, was able to be synergized with PD-1 blockade in increasing anti-tumor immunity among those mice expressing human CD27. PD-1/L1 blockade and anti-CD27 maximally enhanced the levels of Myc protein and a Myc-regulated program of gene expression. Both CD25 and phosphorylated Stat5 (pY694) which is a main IL-2 signaling downstream mediator, are synergistically elevated by PD-1/L1 blockade andanti-CD27. This provides a potential process for co-operative preservation of Myc through combination treatment and suggest that despite increase in IL-2 by anti-CD27, both anti-CD27 and PD-1 blockade are needed to magnify its downstream signaling and capture. Suboptimal invigoration of T-cell in patients who suffer from cancer and are treated with PD-1 checkpoint blockers is enhanced through the combination of CD27 agonism and PD-1 blockade and provides mechanistic insight into how these techniques co-operate with each other to activate CD8+ T cell (Buchan et al., 2018[10]). Ahrends et al. found that the way CD4+T cell helped to optimize CTL response to a DNA vaccine that was made to fight the tumors that expressed human papillomavirus (HPV). CD4+ T cell assisted the optimization of the CTL response by co-stimulating CD27/CD70. Significantly, implementation of an agonistic CD27 antibody significantly replaced helper epitopes to enhance memory and primary CTL responses, acting directly on CD8^+^ T cells. The vaccine turned to be more efficient by CD27 agonism without helper epitopes, and it was more efficient the combination of CTLA-4 blockade and PD-1. When CD27 agonism was combined with CTLA-4 blockade it promoted the priming of CTL induced by vaccine and the infiltration of tumors. However, only when CD27 agonism was combined with PD-1 blockade it turned to be effective at eradicating tumors, thus fully retaining the impact of CD4+ T cell help on the efficacy of vaccine. The sole PD-1 blockade did not leave effect on the priming of CTL or infiltration of tumor, thus these findings suggested that it co-functioned with the help of CD4+ T cell by reducing suppression of CTL in the tumors. Tregs are not stimulated by CD27 agonism or helper epitope inclusion and also the efficiency of vaccine was promoted by CD27 agonism in the presence of CD4+ T cell help. These results provide us with preclinical reasons in applying CD27 agonist antibodies, either in single form or in combination with PD-1 blockade, in improving the efficiency of cancer vaccines and in general terms, the immunotherapy (Ahrends et al., 2016[1]). Wei et al. tried to answer whether anti-CD27 monoclonal antibody can promote the antitumor properties of a DC-based vaccine in mice with prostate malignancies. Anti-CD27 antibody and RM-1 (mouse prostate cancer cell line) tumor lysate-pulsed DCs, in combination, greatly promoted the proliferation and activity of T-cells, and remarkably declined the growth of tumor in comparison to monotherapy with RM-1 tumor lysate-pulsed DCs or anti CD27 antibody (Wei et al., 2015[99]). These findings conclude that, by improving T-cell proliferation and activity, combined treatment can enhance antitumor efficacy (Table 3(Tab. 3); References in Table 3: Ahrends et al., 2016[1]; Buchan et al., 2018[10]; French et al., 2007[22]).

### GITR 

Glucocorticoid-Induced TNFR-Related protein (GITR or TNFSFR18, or CD357) belongs to the TNFR family. It is originally found in T-cell hybridomas of murine who received dexamethasone. Engagement of TCR in CD8^+^ and CD4^+ ^T cells induces its expression (French et al., 2007[22]). Its expression happens at low levels on resting CD4^+^ and CD8^+ ^T cells, 24-72 hours after TCR engagement, it is up-regulated and for several days, its expression remains on the lymphocyte surface (Gurney et al., 1999[29]). On the contrary, Tregs constitutively express GITR, where, as believed, GITR exerted an inhibitory activity on suppressive mechanisms of Tregs (Gurney et al., 1999[29]). In addition, it has been discovered that GITR expression happens on NK cells, basophils, macrophages, eosinophils, and B cells, specifically when it is activated. GITR Ligand (GITRL), similar to OX40L, is remarkably expressed on endothelial cells and activated APCs (Schaer et al., 2012[84]). When ligation happens, downstream signaling of GITR is originated by a complex comprising of two TRAF2 proteins and a TRAF5 resulting in the activation of MAPK and NF-κβ pathways (Snell et al., 2010[89]). The co-stimulation mediated by GITR ultimately relatively increases the proliferation of T-cells and effector functions due to the up-regulation of IL-2, IL-2Rɑ, and IFNɣ (Ronchetti et al., 2004[81]). Ligation of GITR protects T cells from Activation-Induced Cell Death (AICD) which in turn, results in an increase in the number of memory T cells. It has been revealed that GITR stimulation has an anti-tumor effect in various tumor models with an agonist anti-mGITR antibody (rat monoclonal DTA-1) or GITRL manipulation (Ronchetti et al., 2004[81]). Different processes may lead into the modulation of GITR anti-tumor effects. Probably the best explained mechanism is the function of GITR agonists in the abrogation of T-effector cell suppression by Tregs (Stephens et al., 2004[90]; Cohen et al., 2006[15]). Some studies have reported that GITR agonists enhance Teff function in the TME by straightly targeting Tregs impairing, the expression of FoxP3s by Tregs and as a result, abrogation of Tregs suppressive action, or by targeting antigen-specific CD8^+^ T, enhancing its resistance to Tregs suppression. It has been recently revealed that anti-GITR mAb (DTA-1) may straightly deplete intratumor Tregs through the activation of myeloid cells and via FcγRs as a part of its mechanism (Coe et al., 2010[14]). Wang et al. reported that the activation of co-stimulatory pathways to cause powerful activation of T cell probably improves the efficacy of checkpoint inhibition and leads into the lasting antitumor responses. They performed single-cell RNA sequencing of over 2000 tumor-infiltrating CD8+ T cells in mice subjected to PD-1 and GITR antibody combination therapy and concluded, that the effector function of expanded CD8+ T cells is synergistically enhanced by maintaining the balance between the key homeostatic regulators CD226 and the T cell immune receptor containing Ig and ITIM domains (TIGIT), which consequently contributes to a strong survival performance. The dysfunction of CD8^+^ T cell was declined by this combination therapy and it caused a highly proliferative precursor effector memory T cell phenotype in a CD226-dependent manner. The inhibition of PD-1 saved the activity of CD226 through the prevention of PD-1-Src homology region 2 (SHP2) dephosphorylation of the CD226 intracellular domain, while GITR agonism lowered down the expression of TIGIT. The same technique can be used in future clinical trials on cancer immunotherapy to unveil molecular pathways that can drive powerful antitumor responses (Wang et al., 2018[98]). Villarreal et al. concluded that co-administration of aGITR and aPD-1 mAbs when combined with a peptide vaccine (Vax) on mice with developed tumors greatly postponed the growth of tumor and caused total regression in almost 50 % of the mice. This response was related to the promoted expansion and functionality of robust Ag-specific polyfunctional CD8^+^ T cells, generation of memory T cells, and declined Tregs. Regression of tumors was related to the expansion of tumor-infiltrating antigen-specific CD8^+^ effector memory T cells because the depletion of these cells notably declined the efficiency of the triple combination of Vax/aGITR/aPD-1. These results conclude that when dual aGITR/aPD-1 is combined with cancer vaccines can be considered as novel approach in fighting against poorly-immunogenic tumors (Villarreal et al., 2017[95]). Boczkowski et al. studied the new strategy of transfecting DCs with mRNA encoding the light and heavy chain of the anti-GITR mAb. Inducing notably promoted tumor immunity through vaccination with a mixture of tumor antigen-presenting DC and anti-GITR-secreting DC was illustrated in their study. This increase was proportional to the one observed with systemically delivered mAb together with the antigen-presenting DC. More significantly, when anti-GITR was delivered applying RNA-transfected DC, no evidence on autoimmune hypopigmentation in mice with no tumor was observed. Moreover, they reported the promoted induction of cytotoxic T-lymphocyte responses that was solely discerned when the antigen-presenting and antibody-secreting DC was injected concurrently at the similar place (Table 4(Tab. 4); References in Table 4: Coe et al., 2010[14]; Villarreal et al., 2017[95]; Wang et al., 2018[98]). In order to depict the extensive utilization of the approach, the researchers depicted that transfected DC with mRNA encoding GITR-ligand/Fc fusion protein was also an efficient tumor vaccine adjuvant (Boczkowski et al., 2009[7]).

### ICOS

The specific T cell co-stimulatory molecule that belongs to CD28/CTLA-4 family and is constrictively expressed by CD4 T cells is called ICOS. It can co-stimulate the proliferation and cytokine production. ICOS has up-regulated levels in activated T lymphocytes, mainly at post-utilization of anti-CTLA-4 therapies. Its expression can be applied as a biomarker for the indication of anti-CTLA-4 agents that bind to their target (Sanmamed et al., 2015[83]). Elevated expression of ICOS on circulating T cells after using ipilimumab is related to the improved clinical results. ICOS seems to be a less powerful pathway in comparison to other immunotherapy techniques principally due to the predominant expression of CD4 (Fan et al., 2014[19]; Harvey et al., 2015[30]). Nevertheless, when utilized together with other strategies, especially when combined with CTLA-4 blockade it contributes to a robust synergistic effect which are attributed to the increase in ICOS expression post- anti-CTLA-4 therapy (Table 5(Tab. 5); References in Table 5: Burlion et al., 2019[11]; Carrell et al., 2018[12]; Harvey et al., 2015[30]; Sanmamed et al., 2015[83]; Zamarin et al., 2017[105]).

Fan et al. concluded that the combination of CTLA-4 blockade and ICOS engagement by tumor cell vaccines, engineered to express ICOS ligand, qualitatively and quantitatively increased antitumor immune responses and considerably promoted the rejection of developed prostate and melanoma cancer in mice (Fan et al., 2014[19]). Zamarin et al. suggested that intertumoral treatment with Newcastle Disease Virus (NDV), combined with the activation of innate immunity, caused up-regulated the expression of T-cell co-stimulatory receptors, with the ICOS to be significant. By engineering a recombinant NDV-expressing ICOS ligand (NDV-ICOSL), they found ICOS as a direct target in the tumor. Intertumoral application of NDV-ICOSL in bilateral flank tumor models caused increased infiltration of activated T cells in virus-injected and remote tumors resulted in a satisfactory rejection of the tumors when of the combinative form of NDV-ICOSL and systemic CTLA-4 blockade was utilized. These results put emphasize on the fact that intertumoral immunomodulation with an oncolytic virus, that adequately expresses the selected ligand, can be an efficient approach making immune checkpoint blockade to be systematically efficient (Zamarin et al., 2017[105]). Parallel to this finding Carrell et al. studied the potentiality of ICOSL in increasing the immunogenicity of adenoviral-based vaccination targeting the unglycosylated MUC1 peptide antigen. In transgenic mice that recognized human MUC1 as a self-antigen, vaccination hampered immunotolerance and caused strong MUC1-specific immunity. Vaccination enhancment with ICOSL produced a bipolar Th17/Th1 effector profile that is characterized by enhanced production of MUC1-specific IL-17A and increased Orphan nuclear receptor γt (RORγt) expression in CD4^+^ but not CD8^+^ T cells that mainly expressed IFNγ/IL-2 and T-bet. Th17 cells maintenance and polarization established ICOSL augmented vaccination, with elevated levels of IL-17A and RORγt that was identified in CD4^+^ T cells up to 10 months after first immunization. Moreover, MUC1-specific IgG antibody was considerably increased by ICOSL provision in response to immunization. Signaling of ICOSL severely affected CD4^+^ T cell phenotype changing transcription factors gene expression and regulators of effector function after the immunization. Assistance of ICOSL induces lasting, antigen-specific Th17/Th1-mediated immunity *in vivo* and establishes a vaccination platform to increase CD4^+^ T cell-mediated antitumor immunity and also makes a vital component of an efficient cancer vaccine (Carrell et al., 2018[12]). Burlion et al. depicted that targeting ICOS when combined with chemotherapy can be novel approach to promote tumor immunity in humans. Applying a neutralizing mAb to ICOS which is overexpressed by Treg in human tumors, declined the numbers and proportions of Tregs and promotes the proliferation of CD4^+^ T cell in humanized mice. Furthermore, when anti ICOS mAb was combined with cyclophosphamide it decreased the growth of tumors. This is related to the promoted ratio of CD8s to Tregs. The depletion of human CD8^+^ T cells or of murine myeloid cells slightly influenced the efficiency of combination strategy. These findings suggested that when anti-ICOS mAbs are combined with chemotherapy, it controlled tumor growth in humanized mice and opened up horizons in treating breast cancer (Burlion et al., 2019[11]). Electrochemotherapy is a growing therapeutic approach which has recently been claimed to cause an immunogenic form of cell death. Tremble et al. evaluated the impact of electrochemotherapy when combined with ICOS activation that enhanced the function of formerly activated T cells. When compared to monotherapy (that caused results in any model), in a CT26 primary tumor, 50 % of mice were cured, 100 % of cured mice survived from the tumor rechallenge. In a dual flank CT26 model mimicking secondary disease, 20 % of mice were treated, and 30 % of mice were cured from an aggressively metastatic Lewis Lung Carcinoma. They showed that novel combinative form of electrochemotherapy and the activation of ICOS is capable of inhibiting local and distal growth of tumor and total tumor clearance with long-lasting immunological memory (Tremble et al., 2018[92]).

## Conclusion

Over the last decades, the emergence of immune checkpoint blockade, impressive advances have been made in tumor immunotherapy and treatment of various types of solid tumors including renal, bladder, NSCLC, ovarian, gastric and neck cancers. A close examination of the immune and molecular impacts of PD-1/PD-L1 pathways and CTLA-4 has obviously shown that these checkpoint blockades can restore antitumor immune functions, particularly when the two pathways were targeted simultaneously. However, recent studies have been revealed that even in patients treated with immune checkpoint blockade, some resistance mechanism routinely develops and a significant percentage of patients have failed to benefit from this therapy. Nowadays, combination strategies can be utilized to enhance clinical benefits and minimize adverse toxicities. Checkpoint blockade, agonistic antibodies to stimulatory molecules (e.g., OX40, CD137, CD27, and GITR) enhance the activation of T-cells and, as a result, boosted anti-tumor T cell responses. Though stimulatory molecules failed to generate adequate antitumor immunity in poorly immunogenic tumors, then, treating large and developed tumors remains challenging issue owing to the induction of tumor-specific tolerance and immune-suppression.

Latest investigations have been demonstrated that combinations of ICI with agonists for co-stimulatory molecules can lead to activation and amplification of T cells. Indeed, these combinations targeting both co-inhibitory and co-stimulatory molecules are able to overcome tolerance tumor immunity, stimulate a potent CD8^+^ T cell response and ultimately the regression of tumor. One important issue should be considered, timing and sequence of antibody treatment targeting both inhibitory and co-stimulatory receptors are important for a successful combination therapy. It has been shown that significant increases in therapeutic efficacy are achieved by sequential combination of anti-OX40 followed by anti-PD-1 (not reversely). Combinations of different TNF receptor superfamily (TNFRSF) members have been efficiently examined in preclinical models. In addition to checkpoint inhibition, different approaches have been suggested to fulfill the anti-tumor activity of the stimulatory molecules such as radiotherapy, vaccination, chemotherapeutics, and more. The combination of stimulatory molecules and the chemotherapeutics can initiate tumor regression and cause a potent anti-tumor T cell response. Some experiments established that administrating of antibodies to stimulatory molecules at the time of resection can prevent local recurrence of the disease, however, at the time of utilization in conjunction with radiation therapy, it prolonged survival. But there are concerns due to the translation of these methods to clinical regarding the toxicity profile of agonist immunostimulatory monoclonal antibodies (ISMAB). Consequently, various approaches should be suggested on more exclusive delivery of antibodies to the tumor lesion to overcome these limitations. 

## Notes

Ramin Pourakbari and Farnaz Hajizadeh contributed equally as first author.

## Conflict of interest

The authors declare no conflict of interest.

## Authors’ contributions

Ramin Pourakbari and Farnaz Hajizadeh conceived the idea and provided the draft of the manuscript. Forough Parhizkar and Ali Aghebati-Maleki provided inputs for the design and final edition of the article. Sanaz Mansouri participated in literature survey. Leili Aghebati-Maleki critically revised the manuscript. All authors read and approved the final manuscript.

## Funding

This work was supported financially by Research Vice-Chancellor, Tabriz University of Medical Sciences, Tabriz, Iran (Grant number 65564).

## Figures and Tables

**Table 1:**
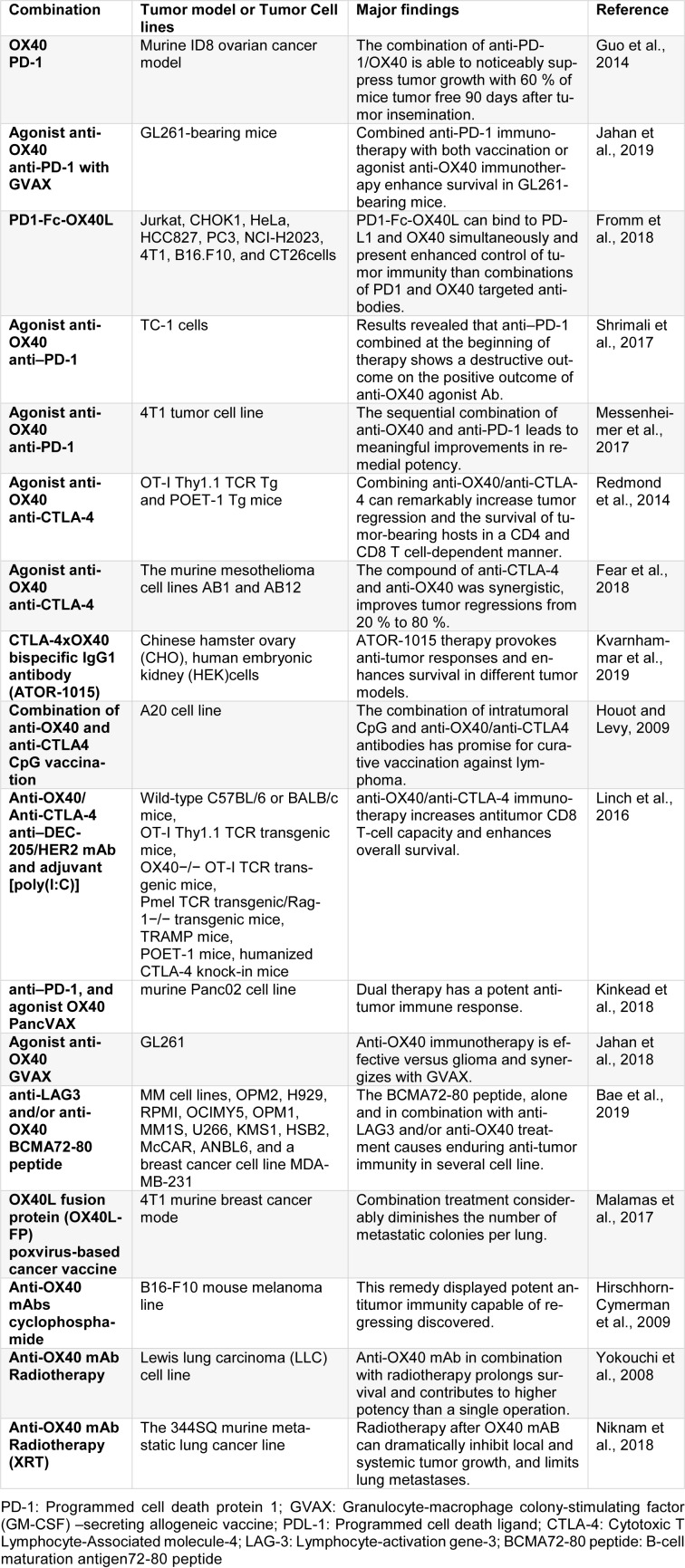
The combination of OX40 with other agents or methods

**Table 2:**
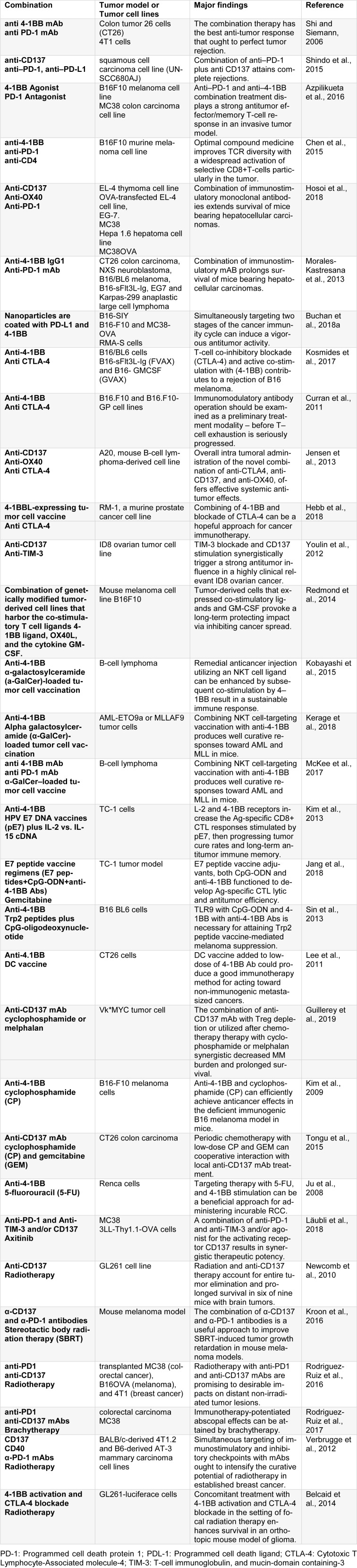
The combination of 4-1BB with other agents or methods

**Table 3:**
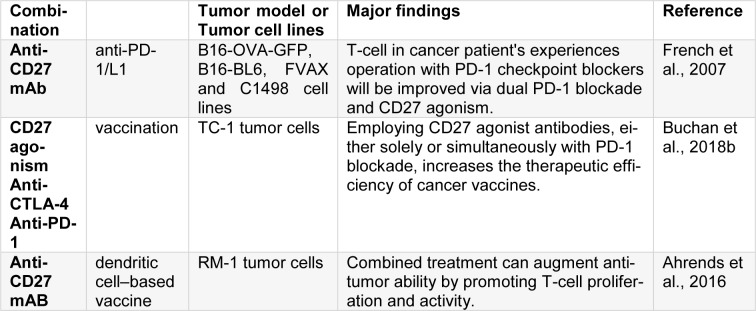
The combination of CD27 with other agents or methods

**Table 4:**
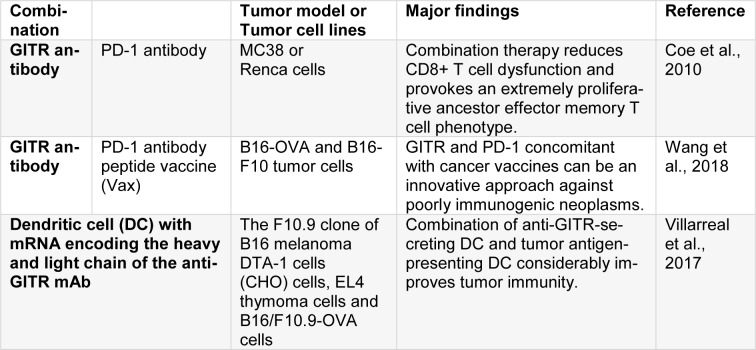
The combination of GITR with other agents or methods

**Table 5:**
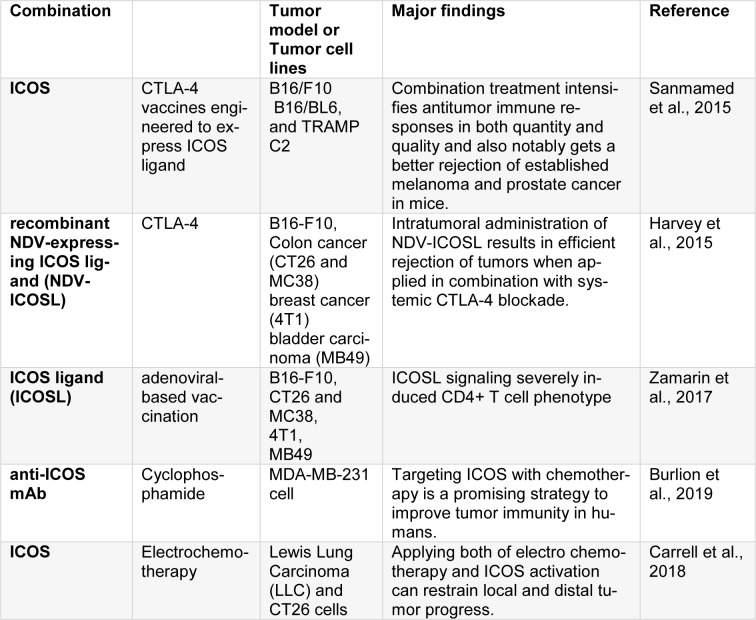
The combination of ICOS with other agents or methods
